# Aberrant use and poor quality of trypanocides: a risk for drug resistance in south western Ethiopia

**DOI:** 10.1186/s12917-017-1327-6

**Published:** 2018-01-05

**Authors:** T. Tekle, G. Terefe, T. Cherenet, H. Ashenafi, K. G. Akoda, A. Teko-Agbo, J. Van Den Abbeele, G. Gari, P.-H. Clausen, A. Hoppenheit, R. C. Mattioli, R. Peter, T. Marcotty, G. Cecchi, V. Delespaux

**Affiliations:** 1 National Animal Health Diagnostic and Investigation Center-Protozoology unit, P.O. Box 8615, Addis Ababa, Ethiopia; 2 Minstry of Livestock and Fisheries, Addis Ababa, Ethiopia; 3 Department of Pathology & Parasitology, Addis Ababa University College of Veterinary Medicine and Agriculture, P.O.Box 34, Bishoftu, Ethiopia; 4 Ecole Inter- Etats des Sciences et Médecine vétérinaires de Dakar, P.O.Box 5077, Dakar, Fann, Senegal; 5 Department of Biomedical Sciences Veterinary Protozoology, Institute of Tropical Medicine, Unit 155 Nationalestraat, B-2000 Antwerp, Belgium; 6 Institute for Parasitology and Tropical Veterinary Medicine, Freie Universitaet Berlin, Robert-von-Ostertag Str. 7-13, 14163 Berlin, Germany; 7 Food and Agriculture Organization of the United Nations, Viale delle Terme di Caracalla, 00153 Rome, Italy; 8 Global Alliance for Livestock Veterinary Medicines (GALVmed), Doherty Building, Pentlands Park, Bush Loan, Edinburgh, EH26 0PZ UK; 9 Veterinary Epidemiology and Risk Analysis - Research and Development (VERDI-R&D), Rue du Gravier 7, 4141 Sprimont, Belgium; 10 Food and Agriculture Organization of the United Nations, Sub-Regional Office for Eastern Africa, Addis Ababa, Ethiopia; 11 Faculty of Sciences and Bio-engineering Sciences, Vrije Universiteit Brussel, Pleinlaan 2, B-1050 Brussels, Belgium

**Keywords:** Diminazene, Isometamidium, Trypanocide, Drug quality assessment, Drug utilization practice, Ethiopia

## Abstract

**Background:**

Trypanocidal drugs have been used to control African animal trypanosomosis for several decades. In Ethiopia, these drugs are available from both authorized (legal) and unauthorized (illegal) sources but documentation on utilization practices and quality of circulating products is scanty. This study looked at the practices of trypanocidal drug utilization by farmers and the integrity of active ingredient in trypanocides sold in Gurage zone, south western Ethiopia. The surveys were based on a structured questionnaire and drug quality determination of commonly used brands originating from European and Asian companies and sold at both authorized and unauthorized markets. One hundred farmers were interviewed and 50 drug samples were collected in 2013 (Diminazene aceturate = 33 and Isometamidium chloride = 17; 25 from authorized and 25 from unauthorized sources). Samples were tested at the OIE-certified Veterinary Drug Control Laboratory (LACOMEV) in Dakar, Senegal, by using galenic standards and high performance liquid chromatography.

**Results:**

Trypanosomosis was found to be a major threat according to all interviewed livestock keepers in the study area. Diminazene aceturate and isometamidium chloride were preferred by 79% and 21% of the respondents respectively, and 85% of them indicated that an animal receives more than six treatments per year. About 60% of these treatments were reported to be administered by untrained farmers. Trypanocidal drug sources included both unauthorized outlets (56%) and authorized government and private sources (44%). A wide availability and usage of substandard quality drugs was revealed. Twenty eight percent of trypanocidal drugs tested failed to comply with quality requirements. There was no significant difference in the frequency of non-compliance between diminazene-based and isometamidium chloride products (*P* = 0.87) irrespective of the marketing channel (official and unofficial). However, higher rates of non-compliant trypanocides were detected for drugs originating from Asia than from Europe (*P* = 0.029).

**Conclusion:**

The findings revealed the presence of risk factors for the development of drug resistance, i.e. wide distribution of poor quality drugs as well as substandard administration practices. Therefore, it is strongly recommended to enforce regulatory measures for quality control of veterinary drugs, to expand and strengthen veterinary services and to undertake trypanocidal drug efficacy studies of wider coverage.

**Electronic supplementary material:**

The online version of this article (10.1186/s12917-017-1327-6) contains supplementary material, which is available to authorized users.

## Background

Ethiopia has the largest livestock population in Africa with 53.9 million cattle, 24.6 million goats, 25.5 million sheep 6.8 million donkeys and 1.9 million horses [1]. Hence, livestock is a significant contributor to economic and social development of the country. Livestock accounts for 15 to 17% and 35 to 49% of the total and agricultural GDP respectively [1]. Unfortunately, the development and intensification of livestock production is hampered by transboundary epizootic diseases such as African animal trypanosomosis (AAT). It is estimated that, should AAT be eliminated in Ethiopia, direct benefits would exceed 800 million USD over a 20-year period [2].

Various efforts to control the disease and the associated economic losses have been directed mainly against the parasite through trypanocidal drugs and against the vector through odour-baited and insecticide- impregnated targets/traps and insecticide-treated cattle [3–5]. The main drugs used for treating the disease, i.e. diminazene aceturate (DA) and isometamidium chloride (ISM) have been on the market for more than half a century and the parasites’ resistance to both trypanocidal drugs is increasing [6].

In Ethiopia, several authors reported prevalence of drug resistance against one or both of the drugs (DA and ISM) in several AAT-affected areas [7–12]. It is believed that the emergence of drug resistance is linked to bad handling and utilization practices as well as to poor drug quality [3, 13, 14], which severely reduces the effectiveness of chemotherapy. Drug resistance to ISM is more widespread than to DA [15], but multiple drug resistance is being increasingly reported from different parts of Africa [16, 17].

None of the previous studies has assessed the quality of trypanocidal drugs in Ethiopia. The present study aimed to fill this knowledge gap. The study was initiated as part of a joint action for trypanocidal drug quality control in Africa whose partners include the Global Alliance for Livestock Veterinary Medicine (GALVmed), the Food and Agriculture Organization of the United Nations (FAO), the International Federation of Animal Health (IFAH), the International Atomic Energy Agency (IAEA) and the Trypanosomosis Rational Chemotherapy (TRYRAC) project, funded by the European Commission. The specific objectives of this study were to assess trypanocidal drug utilization practices in south-western Ethiopia and evaluate the quality of diminazene aceturate- and isometamidium chloride-based brands.

## Methods

### Study sites

**Fig. 1:**
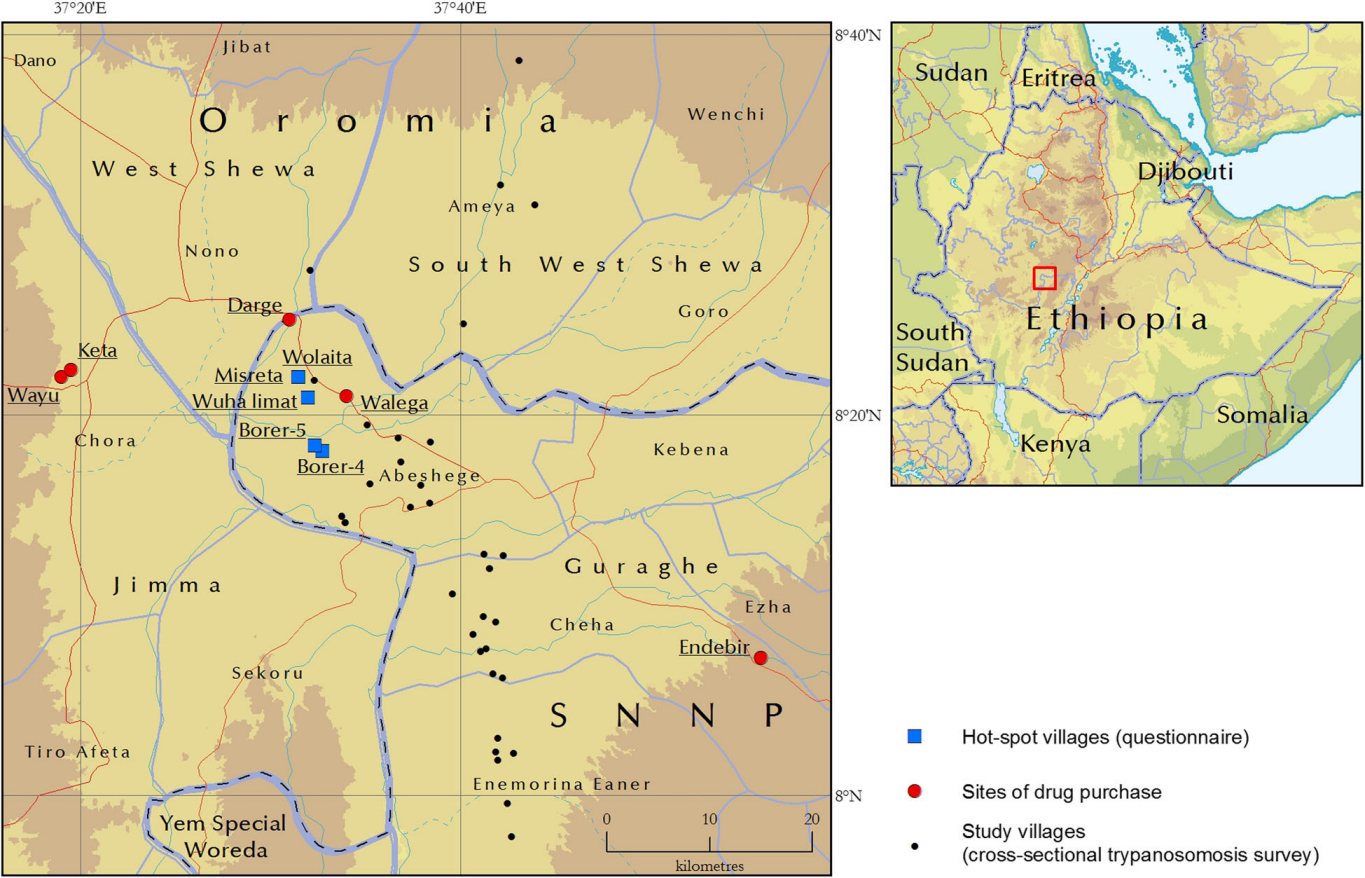
Map of the study area showing sampling sites for crossesctional study (black dotes), trypanocidal drug sampling sites (red bullets) and the five hot spot villages where drug treatment trial and questionnaire survey were done (blue bullets). Bullets for Wolaita and Misreta villages are overlaping

The study was conducted in south-western Ethiopia, at the boundary between the Southern Nations, Nationalities and Peoples’ Region (Gurage zone) and Oromia Region (Jimma zone). In these areas, three species of tsetse flies are present (*G. pallidipes, G. fuscipes fuscipes, G. morsitans submorsitans*) [18]. According to information obtained from local authorities, there were 124 tsetse- and trypanosome-affected villages in the study areas. After randomly assessing 40 villages among the 124, five villages with trypanosome prevalence ≥10% were identified for the questionnaire survey: Borer 4, Borer 5, Misreta, Wolaita and Wuhalimat. Trypanocidal drugs were collected from authorized (veterinary clinics and drug stores) and unauthorized markets of the selected areas (Fig. 1) and wholesalers supplying the areas. The drugs were analysed at the Veterinary Drug Control Laboratory (LACOMEV) in Dakar, Senegal.

### Questionnaire survey

A structured questionnaire was used to collect data on the practice of trypanocidal drug usage amongst farmers in the five selected study villages. A total of 100 farmers selected by systematic random sampling technique (20 farmers in each village) were interviewed. The respondents were selected from farmers who were voluntarily presenting their cattle for trypanosomosis screening during the study period.

### Purchase of trypanocidal products for quality assessment

Fifty samples of trypanocidal drugs were collected from different vendors (authorized and unauthorized) and wholesalers in October 2013. The sampling points were purposely selected for consistency with the drug supply facilities mentioned in the questionnaire survey. Sampling points included authorized markets (Veterinary Clinics, pharmacies and wholesalers) and unauthorized sources (open markets) (Table 1). The drugs were sealed in plastic bags identified by a unique number. Information such as trade name, manufacturer, origin, date of manufacture, expiry date, and place of purchase were recorded. To ensure sufficient quantity, each sample contained at least 5–10 sachets depending on the content per sachet (Five for the 23.6 g DA and 1 g Ism, 10 for the 2.36 g DA and 125 mg ISM) to ensure sufficient sample for analysis.

**Table 1:** Sources of trypanocidal drugs used for quality analysis (drug samples were collected from nearby authorized vendors and suppliers as well as open markets/illegal sources)

	Wholesaler	Governmental clinic^a^	Private veterinary pharmacy^a^	Open market^b^	Total
ISM	5	3	2	7	17
DA	5	2	8	18	33
Total	**10**	**5**	**10**	**25**	**50**

^a^authorized, ^b^unauthorized

*ISM* Isometamidium chloride, *DA* Diminazene aceturate

### Assessment of trypanocidal drug quality

Drug quality was assessed by (i) galenic tests, (ii) identification and (iii) measurement of concentration of the respective active ingredient according to standard operating procedures prepared by GALVmed, FAO and IFAH in collaboration with Manchester Metropolitan University and IAEA [19]. The galenic testing included pH measurement, solubility/limpidity of solutions prepared from DA granules or ISM powder according to the manufacturers’ recommendations. The pH was measured using a Metler MP 230 pH meter with a pH between 4 and 7 considered as compliant. The limpidity of solutions was assessed visually with the naked eye for the absence of visible solid particles Identification and concentration of the active ingredient were assessed using high performance liquid chromatography (HPLC) [19]. Each sample was simultaneously measured with a reference standard. The standards for diminazene and isometamidium were manufactured by VETOQUINOL (Paris, France) and CEVA (Libourne, France), respectively [19] and were provided to LACOMEV by the consortium GALVmed/FAO/IAEA/IFAH. They were stored in a refrigerator at 4° +/− 1 °C until use.

Trypanocide samples were dissolved in ultrapure water for DA and 25% acetonitrile in ultrapure water for ISM to obtain a solution of 0.1 mg/ml of active ingredient. The solution was poured into vials and introduced into the HPLC machine that was programmed to automatically conduct the process of identification and concentration measurement. The mobile phase for the analysis of DA used a mix of 10% methanol, 10% acetonitrile and 80% ammonium formate buffer (20 mM, pH 4.0). Similarly, the mobile phase for ISM used 25% acetonitrile and 75% ammonium formate buffer (50 mM, pH 2.8). A Water Kromasil C18® HPLC column (150 × 4.6 mm, 5 μm - AkzoNobel, Separation Products, Bohus, Sweden) was used to run the test. The procedure was performed twice with a maximum acceptable divergence between analyses of 2%. In case of higher divergence, the procedure was repeated until it fell within the 2% threshold. For DA, a measured concentration within ±10% variation from the manufacturers’ label claim was considered as compliant [20]. For ISM, the following criteria were used to declare compliance: (i) presence of the four isomers (I, II, III, IV), (ii) a proportion of isomer I (principal component) equal to or greater than 55%, (iii) a proportion of isomers II, III and IV equal to or less than 40% and (iv) a proportion of the four isomers falling between 95 and 102%. All along the quality assessment process, names of pharmaceutical companies producing the drugs were kept anonymous.

### Statistical analysis

Confidence intervals of proportions were calculated assuming binomial distributions of the variables. For drug quality compliance study, a logistic regression was employed. The response was drug compliance whereas binary explanatory variables were the drugs (DA/ISM), the marketing channel (official/unofficial) and the origin of the drugs (Asia/Europe). Non-significant explanatory variables (*p* > 0.05) were removed from the model. In the event that a category contained no observation, the exact test was used to evaluate the significance of the difference and the exact method was used to calculate the confidence interval.

### Ethical considerations

The objectives of this study were well explained to all selected farmers and those who expressed their consent to participate in the questionnaire survey were recruited. The identity of study participants and data on their livestock population were kept confidential.

## Results

### Trypanocidal drug utilization practices

For all respondent farmers communal or free grazing is the predominant livestock management practice in the study villages and trypanosomosis was ranked as biggest animal health constraint.

**Fig. 2:**
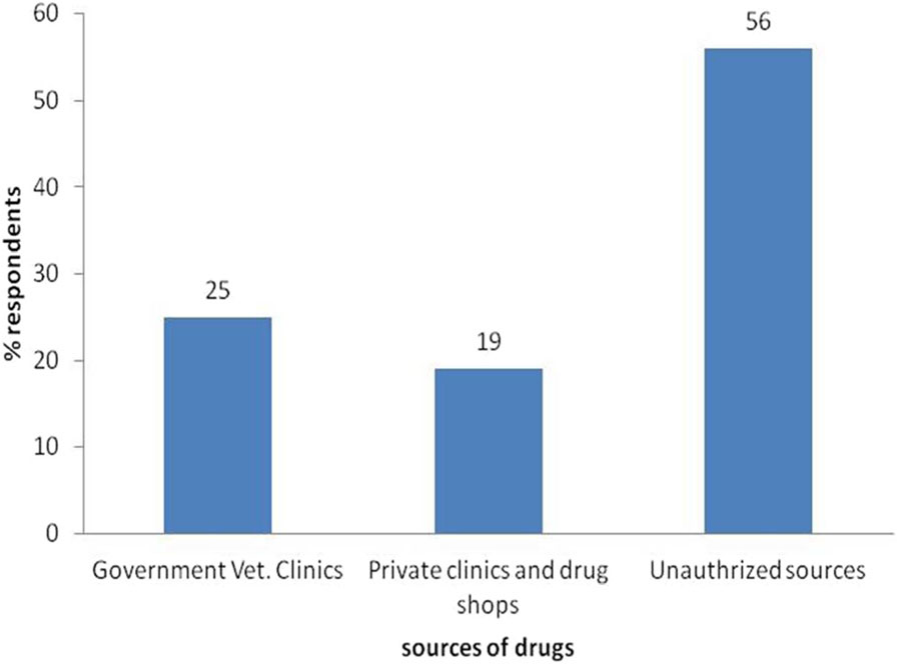
Questionnaire survey response on the source of trypanocidal drugs for treatment of cattle in the study areas

**Fig. 3:**
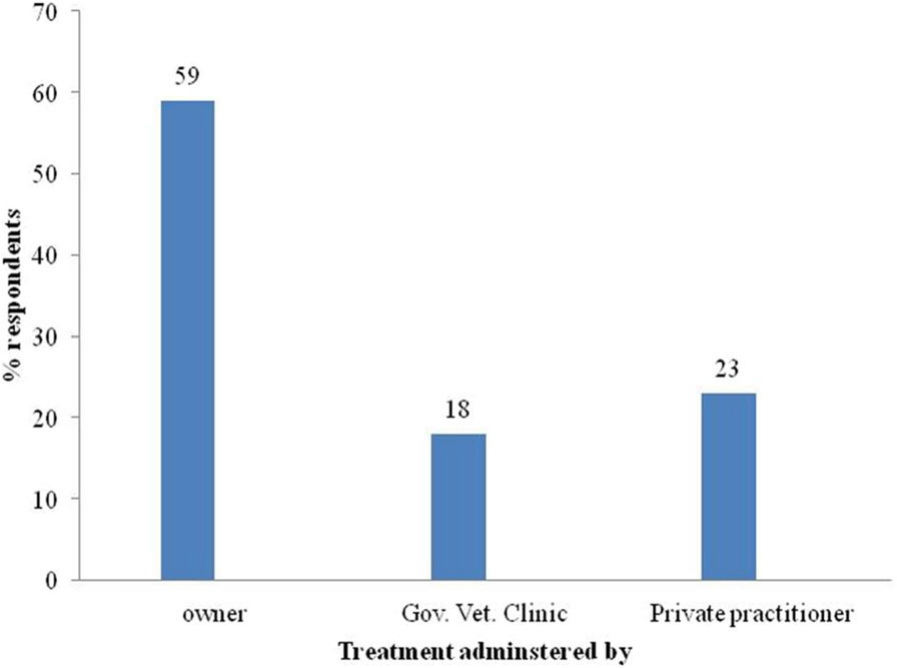
Questionnaire survey response on the administration (injection) of trypanocidal drugs to cattle in the study area

**Fig. 4:**
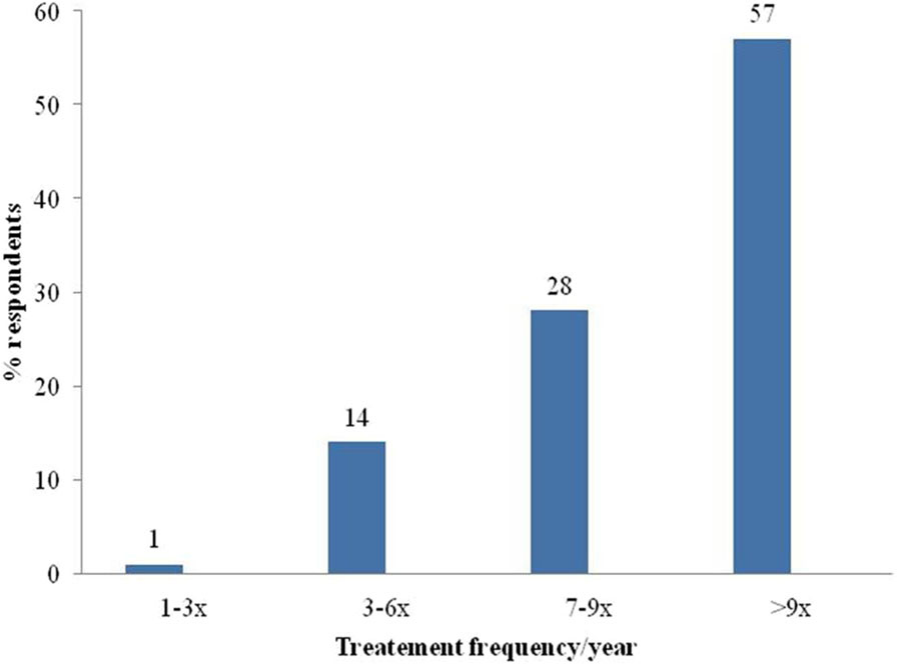
Annual trypanocidal drug treatment frequency per individual cattle based on the questionnaire survey response

For the control of trypanosomosis, all respondents reported to depend mainly on the two trypanocidal drugs diminazene aceturate and isometamidium chloride rather than other control methods (Additional file 1: Annex I). The majority of respondents in all villages (56%; 95% CI = 45.7–65.9%) get trypanocidal drugs from unauthorized markets (Fig. 2). Diminazene aceturate was the preferred drug (79%) over ISM (21%). Respondents explained that diminazene was cheaper and available from all sources in single dose/sachet. On the other hand, significant numbers of farmers in the study villages (59%; 95%; CI = 48.7–68.7%) administer trypanocidal drugs by themselves or through family members (Fig. 3) and about 85% of them (95% CI = 76.5–91.4%) indicated that they treat their animals seven or more times per year (Fig. 4). All respondents perceived treatment failures as common. In this respect, 58% of the interviewed farmers believe that treatments were more likely to be successful when the drugs are sourced from government veterinary clinics and authorized private sources than from unauthorized open markets.

### Trypanocidal drug quality

**Fig. 5:**
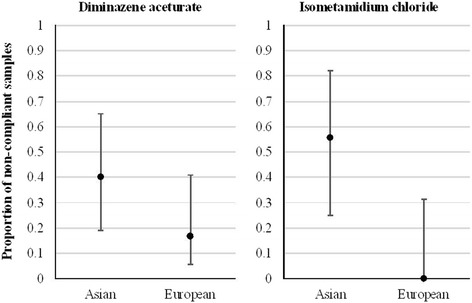
Proportions of non-compliant samples according to continental origin (Asian and European) of the drugs with 95% confidence intervals (based on a logistic regression using drug, origin and the interaction between them as explanatory variables; the exact method was used for categories with no observations)

Of the 50 trypanocidal drug samples collected, 26 were from European companies and 24 from Asian companies. The samples represent 19 different trade names (9 for ISM and 10 for DA). Overall, result showed that 28% (14/50) of the drugs were non-compliant due to insufficient active ingredient detected by HPLC. The difference in non-compliance between the two drugs was not significant (*P* = 0.87 in univariate model), being 27.3% for DA and 29.4% for ISM. Also, clients of authorized and non-authorized markets are at similar risk of purchasing non-compliant trypanocides (Table 2; *P* = 0.53 in univariate model). On the other hand, logistic regression analysis shows that the proportion of non-compliant drugs was significantly more important amongst Asian drugs (*P* = 0.01 in multivariate model) especially for ISM (interaction not tested since all European ISM samples were found compliant) (Fig. 5). Among the 14 non-compliant samples, nine were from Indian origin (four brands) and two were from UK (one brand). The correlation between marketing channel (official and unofficial) and trypanocidal origin (Asia and Europe) is not significant (*P* = 0.26 in a Chi Square test).

**Table 2:** Proportions of non-compliant samples according to the marketing channel and drug type

Molecule	Illegal(unauthorized)		Legal(authorized)	
	Complies	Not complies	Complies	Not complies
Diminazene Aceturate	13	5	11	4
Isometamidium chloride	4	3	8	2

Number of observations

## Discussion

Several control approaches are available to overcome the impacts of AAT [21]. Treatment by the use of trypanocidal drugs such as diminazene aceturate and isometamidium chloride is oftentimes the only method available to farmers for containing the disease in many parts of Ethiopia. In this respect, there is a growing risk of drug resistant trypanosomes as already reported by previous studies [7–12]. In this study, it was observed that risk factors for drug resistance such as the presence of unofficial drug sources and frequent treatments are widespread in the study areas. Diminazene aceturate was reported to be the most frequently used compared to isometamidium chloride. This is believed to expose the drug to more risk of trypanocidal resistance. Inadequate veterinary services and the higher price of drugs from legal sources might have contributed for the greater frequency of respondents opting for unauthorized/illegal sources to easily access trypanocidal drugs. A similar report was documented by Zewdu et al. [22] where annual treatment frequencies range between one and 12 injections/animal and where a significant number of farmers directly gave injections without counseling from a veterinarian. According to Uilenberg [14] and Holmes et al. [3], a high number of annual trypanocidal treatments is suggestive of drug resistance in a given area. Therefore, the high frequency of trypanocidal treatments (more than six times per year), access to drugs from unauthorized sources and the practice of treating animals by untrained personnel is likely to increase the risk of trypanocidal resistance in the study areas and neighbouring localities.

Quality assessment of trypanocides is a prerequisite to ensure better management of trypanosomosis and the prevention of drug resistance [23]. Therefore, trypanocidal drugs were bought anonymously in order to avoid attracting the attention of vendors, who otherwise might have denied access to the products. The 28% of non-compliance observed in this study was below the 71.4% reported in Ivory Coast [24], 100% in Cameroon [25], 70% in Senegal [26], 40% in Togo [27] and 42.3% observed in Burkina Faso [28]. However, since the drugs were supplied by big companies who are national distributers, non-compliant products pose a serious threat to successful AAT treatments countrywide.

The non-compliant veterinary products identified by this study were found in both authorized and unauthorized markets which is different from reports from Togo and Mali [27–29] where non-conform drugs were significantly more often found on unauthorized markets. The non-compliance may be attributed to poor storage conditions and the doubtful sources of supply in this market channel. The 2012 Proclamation for Veterinary Drugs and Feed Administration and Control of Ethiopia capitalizes on regulating “the production, distribution and use of veterinary drugs to ensure safety, efficacy and quality of the products”. It also focuses on “prevention and control of the illegal production, distribution and use of veterinary drugs” [30]. Therefore, the observation of such a significant frequency of non-compliant trypanocides undermines the basic objective of the existing law. Although there are encouraging signs, the existing situation signals the urgent need to enforce the legislative framework at all levels to reduce and prevent illegal marketing and use of veterinary drugs. A similar failure to adequately regulate and control medicinal products for human use has already been reported in the country [31].

Although this study did not demonstrate significant variation in non-compliance between the two drugs, coupled with the high pressure on diminazene due to its short biological half-life and hence more frequent administration [32, 33], this loss of quality would mean a greater risk for the development of resistance. Various brands of trypanocides originating from several countries in Europe and Asia have shown different levels of compliance to drug quality casting doubt on the reputability of some of the manufacturers of veterinary products [27]. In this respect, the detection of non-compliant drugs within samples sourced from wholesalers strongly suggests that some of these drugs might be defective right from their origin and in such case unauthorized vendors in rural areas might have obtained them from legal importers/suppliers. On the other hand, since some of the brand names are identical between both authorized and unauthorised sources, it is also possible that counterfeit drugs are being sold carrying brand names of genuine manufacturers.

The impact of such quality defects in veterinary trypanocidal drug products can be very significant. A lack of efficacy will cause financial harm due to under dosage and residues in the muscle tissue damaging consumers when the concentration is above the prescribed limit [34, 35]. Some specialists [20] quite rightly believe that the circulation of counterfeit drugs in most sub-Saharan African countries inevitably leads to the persistence of animal diseases. This is proof of the urgent need to introduce systematic controls on the supply, distribution and utilization channels for veterinary trypanocidal products in Ethiopia.

## Conclusion

Although the results obtained by this study may not represent trypanocidal drug use of the entire country, they clearly show the effects of poor quality veterinary trypanocidal drugs which are a result of the lack of a proper import control and country administration of veterinary controls in the study area and probably beyond. Unsafe utilization practices and poor handling of the drugs by farmers is also believed to aggravate the situation. Non-compliant trypanocidal drugs are known to compromise the efficacy of treatment and escalate the development of drug resistance, which has already been reported in different parts of Ethiopia. Corrective measures must therefore be taken towards effective implementation of regulatory and legislative frameworks, quality control of veterinary trypanocidal drugs and improving the coverage and quality of veterinary services and regular monitoring of trypanocidal drug efficacy.

## Additional files

Additional file 1: Annex 1. Questionnaire survey data: trypanocidal drug utilization practices, perception of risks of bovine trypanosomosis and efficacy of existing trypanocidal drugs. (DOCX 42 kb)

## Abbreviations

AAT: African animal trypanosomosis
DA: Diminazene aceturate
FAO: Food and Agriculture Organization
GALVmed: Global Alliance for Livestock Veterinary Medicine
GDP: Gross domestic product
HPLC: High performance liquid chromatography
IAEA: International Atomic Energy Agency
IFAH: International Federation for Animal Health
ISM: Isometamidium
LACOMEV: Laboratoire de Contrôle des Médicaments Vétérinaires
TRYRAC: Trypanosomosis Rational Chemotherapy
