# Supplementary material for: Aberrant use and poor quality of trypanocides: a risk for drug resistance in south western Ethiopia
Source: BMC Vet Res. 2018 Jan 5;14:4. doi: 10.1186/s12917-017-1327-6 (PMC5755418; doi:10.1186/s12917-017-1327-6)
Supplement: Supplementary file 1 — Questionnaire survey data: trypanocidal drug utilization practices, perception of risks of bovine trypanosomosis and efficacy of existing trypanocidal drugs. (DOCX 42 kb) [file 12917_2017_1327_MOESM1_ESM.docx]

| **Annex I** | | | | | | | | | | | |  |  | |
| --- | --- | --- | --- | --- | --- | --- | --- | --- | --- | --- | --- | --- | --- | --- |
| Respondent ID | Village | cattle grazing practice | Is trypanosomosis a threat? | rank of tryps Vs other diseases | main disease management method | preferred drug | drug source | who injects the drugs? | treatment freq. /anim/year | is treatment failure common? | treatment efficacy Vs drug source |  | Keys | |
| 1 | 1 | communal | Yes | 1st | Treatment | 2 | 3 | 1 | 4 | Yes | 3 |  | villages |  |
| 2 | 1 | communal | Yes | 1st | Treatment | 2 | 3 | 1 | 4 | Yes | 1 |  | 1 | Borer-4 |
| 3 | 1 | communal | Yes | 1st | Treatment | 2 | 1 | 2 | 1 | Yes | 1 |  | 2 | Borer-5 |
| 4 | 1 | communal | Yes | 1st | Treatment | 2 | 3 | 3 | 4 | Yes | 1 |  | 3 | WuhaLimat |
| 5 | 1 | communal | Yes | 1st | Treatment | 2 | 3 | 3 | 4 | Yes | 2 |  | 4 | Misreta |
| 6 | 1 | communal | Yes | 1st | Treatment | 2 | 3 | 1 | 4 | Yes | 1 |  | 5 | Wolaita |
| 7 | 1 | communal | Yes | 1st | Treatment | 2 | 3 | 1 | 4 | Yes | 3 |  |  |  |
| 8 | 1 | communal | Yes | 1st | Treatment | 2 | 3 | 1 | 3 | Yes | 1 |  | Drug adminstered by |  |
| 9 | 1 | communal | Yes | 1st | Treatment | 2 | 3 | 2 | 4 | Yes | 2 |  | 1 | self |
| 10 | 1 | communal | Yes | 1st | Treatment | 2 | 1 | 2 | 3 | Yes | 1 |  | 2 | Gvt. Vet.Clinician |
| 11 | 1 | communal | Yes | 1st | Treatment | 2 | 1 | 2 | 2 | Yes | 1 |  | 3 | Pvt. Clinician |
| 12 | 1 | communal | Yes | 1st | Treatment | 2 | 3 | 1 | 3 | Yes | 3 |  |  |  |
| 13 | 1 | communal | Yes | 1st | Treatment | 2 | 1 | 1 | 2 | Yes | 1 |  | Drug source |  |
| 14 | 1 | communal | Yes | 1st | Treatment | 2 | 3 | 1 | 4 | Yes | 1 |  |  |  |
| 15 | 1 | communal | Yes | 1st | Treatment | 2 | 3 | 1 | 4 | Yes | 2 |  | 1 | Gvt. Vet. Clinic |
| 16 | 1 | communal | Yes | 1st | Treatment | 1 | 3 | 1 | 4 | Yes | 3 |  | 2 | PVt. Drug store |
| 17 | 1 | communal | Yes | 1st | Treatment | 2 | 1 | 2 | 3 | Yes | 1 |  | 3 | open market |
| 18 | 1 | communal | Yes | 1st | Treatment | 2 | 2 | 1 | 4 | Yes | 2 |  | Treat. Freq./anim/year | |
| 19 | 1 | communal | Yes | 1st | Treatment | 2 | 3 | 1 | 2 | Yes | 1 |  | 1 | 1 to 3 |
| 20 | 1 | communal | Yes | 1st | Treatment | 1 | 1 | 1 | 3 | Yes | 1 |  | 2 | 4 to 6 |
| 21 | 2 | communal | Yes | 1st | Treatment | 2 | 3 | 3 | 4 | Yes | 3 |  | 3 | 7 to 9 |
| 22 | 2 | communal | Yes | 1st | Treatment | 1 | 1 | 3 | 2 | Yes | 1 |  | 4 | >9 |
| 23 | 2 | communal | Yes | 1st | Treatment | 1 | 2 | 1 | 4 | Yes | 1 |  | Efficacy |  |
| 24 | 2 | communal | Yes | 1st | Treatment | 1 | 2 | 1 | 4 | Yes | 2 |  | A | yes |
| 25 | 2 | communal | Yes | 1st | Treatment | 2 | 2 | 1 | 3 | Yes | 2 |  | B | No |
| 26 | 2 | communal | Yes | 1st | Treatment | 2 | 3 | 1 | 4 | Yes | 3 |  | Source that provide more effective drug |  |
| 27 | 2 | communal | Yes | 1st | Treatment | 2 | 3 | 2 | 2 | Yes | 1 |  | 1 | Gov. vet. Clinic |
| 28 | 2 | communal | Yes | 1st | Treatment | 2 | 3 | 2 | 3 | Yes | 1 |  | 2 | PVt. Drug store |
| 29 | 2 | communal | Yes | 1st | Treatment | 2 | 2 | 3 | 4 | Yes | 2 |  | 3 | open market |
| 30 | 2 | communal | Yes | 1st | Treatment | 2 | 1 | 3 | 4 | Yes | 1 |  |  |  |
| 31 | 2 | communal | Yes | 1st | Treatment | 2 | 1 | 1 | 2 | Yes | 1 |  |  |  |
| 32 | 2 | communal | Yes | 1st | Treatment | 2 | 1 | 3 | 4 | Yes | 3 |  | Type of drug |  |
| 33 | 2 | communal | Yes | 1st | Treatment | 2 | 3 | 1 | 3 | Yes | 1 |  | 1 | Brown |
| 34 | 2 | communal | Yes | 1st | Treatment | 2 | 1 | 1 | 4 | Yes | 1 |  | 2 | Yellow |
| 35 | 2 | communal | Yes | 1st | Treatment | 2 | 3 | 1 | 3 | Yes | 2 |  |  |  |
| 36 | 2 | communal | Yes | 1st | Treatment | 2 | 3 | 1 | 2 | Yes | 1 |  | Is trypanosomosis a threat | |
| 37 | 2 | communal | Yes | 1st | Treatment | 2 | 3 | 1 | 4 | Yes | 1 |  |  | 1yes |
| 38 | 2 | communal | Yes | 1st | Treatment | 2 | 3 | 1 | 3 | Yes | 3 |  |  | 2no |
| 39 | 2 | communal | Yes | 1st | Treatment | 2 | 3 | 1 | 4 | Yes | 1 |  |  |  |
| 40 | 2 | communal | Yes | 1st | Treatment | 2 | 3 | 3 | 3 | Yes | 2 |  |  |  |
| 41 | 3 | communal | Yes | 1st | Treatment | 2 | 3 | 3 | 3 | Yes | 1 |  |  |  |
| 42 | 3 | communal | Yes | 1st | Treatment | 2 | 3 | 3 | 4 | Yes | 2 |  |  |  |
| 43 | 3 | communal | Yes | 1st | Treatment | 2 | 3 | 1 | 2 | Yes | 2 |  |  |  |
| 44 | 3 | communal | Yes | 1st | Treatment | 1 | 3 | 3 | 3 | Yes | 1 |  |  |  |
| 45 | 3 | communal | Yes | 1st | Treatment | 2 | 3 | 3 | 4 | Yes | 3 |  |  |  |
| 46 | 3 | communal | Yes | 1st | Treatment | 2 | 2 | 1 | 3 | Yes | 2 |  |  |  |
| 47 | 3 | communal | Yes | 1st | Treatment | 2 | 2 | 3 | 4 | Yes | 2 |  |  |  |
| 48 | 3 | communal | Yes | 1st | Treatment | 1 | 2 | 3 | 2 | Yes | 2 |  |  |  |
| 49 | 3 | communal | Yes | 1st | Treatment | 2 | 1 | 1 | 3 | Yes | 1 |  |  |  |
| 50 | 3 | communal | Yes | 1st | Treatment | 2 | 1 | 1 | 4 | Yes | 1 |  |  |  |
| 51 | 3 | communal | Yes | 1st | Treatment | 2 | 1 | 1 | 3 | Yes | 1 |  |  |  |
| 52 | 3 | communal | Yes | 1st | Treatment | 2 | 1 | 1 | 4 | Yes | 1 |  |  |  |
| 53 | 3 | communal | Yes | 1st | Treatment | 2 | 3 | 3 | 2 | Yes | 1 |  |  |  |
| 54 | 3 | communal | Yes | 1st | Treatment | 2 | 3 | 2 | 4 | Yes | 2 |  |  |  |
| 55 | 3 | communal | Yes | 1st | Treatment | 2 | 3 | 2 | 4 | Yes | 2 |  |  |  |
| 56 | 3 | communal | Yes | 1st | Treatment | 2 | 3 | 2 | 2 | Yes | 1 |  |  |  |
| 57 | 3 | communal | Yes | 1st | Treatment | 2 | 3 | 2 | 3 | Yes | 1 |  |  |  |
| 58 | 3 | communal | Yes | 1st | Treatment | 1 | 3 | 2 | 4 | Yes | 1 |  |  |  |
| 59 | 3 | communal | Yes | 1st | Treatment | 2 | 3 | 2 | 3 | Yes | 2 |  |  |  |
| 60 | 3 | communal | Yes | 1st | Treatment | 2 | 2 | 3 | 4 | Yes | 2 |  |  |  |
| 61 | 4 | communal | Yes | 1st | Treatment | 2 | 2 | 3 | 2 | Yes | 2 |  |  |  |
| 62 | 4 | communal | Yes | 1st | Treatment | 2 | 1 | 1 | 4 | Yes | 2 |  |  |  |
| 63 | 4 | communal | Yes | 1st | Treatment | 2 | 1 | 1 | 4 | Yes | 2 |  |  |  |
| 64 | 4 | communal | Yes | 1st | Treatment | 1 | 3 | 1 | 3 | Yes | 1 |  |  |  |
| 65 | 4 | communal | Yes | 1st | Treatment | 1 | 3 | 1 | 2 | Yes | 1 |  |  |  |
| 66 | 4 | communal | Yes | 1st | Treatment | 1 | 3 | 1 | 4 | Yes | 1 |  |  |  |
| 67 | 4 | communal | Yes | 1st | Treatment | 1 | 3 | 3 | 3 | Yes | 3 |  |  |  |
| 68 | 4 | communal | Yes | 1st | Treatment | 2 | 3 | 3 | 4 | Yes | 2 |  |  |  |
| 69 | 4 | communal | Yes | 1st | Treatment | 1 | 3 | 3 | 3 | Yes | 1 |  |  |  |
| 70 | 4 | communal | Yes | 1st | Treatment | 2 | 3 | 3 | 4 | Yes | 2 |  |  |  |
| 71 | 4 | communal | Yes | 1st | Treatment | 1 | 3 | 3 | 4 | Yes | 1 |  |  |  |
| 72 | 4 | communal | Yes | 1st | Treatment | 2 | 2 | 1 | 4 | Yes | 2 |  |  |  |
| 73 | 4 | communal | Yes | 1st | Treatment | 2 | 2 | 2 | 4 | Yes | 2 |  |  |  |
| 74 | 4 | communal | Yes | 1st | Treatment | 2 | 2 | 2 | 4 | Yes | 2 |  |  |  |
| 75 | 4 | communal | Yes | 1st | Treatment | 2 | 2 | 1 | 4 | Yes | 2 |  |  |  |
| 76 | 4 | communal | Yes | 1st | Treatment | 1 | 1 | 3 | 4 | Yes | 1 |  |  |  |
| 77 | 4 | communal | Yes | 1st | Treatment | 1 | 3 | 3 | 3 | Yes | 1 |  |  |  |
| 78 | 4 | communal | Yes | 1st | Treatment | 2 | 3 | 3 | 4 | Yes | 3 |  |  |  |
| 79 | 4 | communal | Yes | 1st | Treatment | 2 | 3 | 1 | 4 | Yes | 1 |  |  |  |
| 80 | 4 | communal | Yes | 1st | Treatment | 2 | 3 | 2 | 3 | Yes | 1 |  |  |  |
| 81 | 5 | communal | Yes | 1st | Treatment | 2 | 1 | 2 | 4 | Yes | 3 |  |  |  |
| 82 | 5 | communal | Yes | 1st | Treatment | 1 | 1 | 2 | 3 | Yes | 1 |  |  |  |
| 83 | 5 | communal | Yes | 1st | Treatment | 1 | 1 | 2 | 4 | Yes | 1 |  |  |  |
| 84 | 5 | communal | Yes | 1st | Treatment | 2 | 3 | 3 | 4 | Yes | 2 |  |  |  |
| 85 | 5 | communal | Yes | 1st | Treatment | 1 | 2 | 3 | 4 | Yes | 2 |  |  |  |
| 86 | 5 | communal | Yes | 1st | Treatment | 2 | 3 | 3 | 3 | Yes | 1 |  |  |  |
| 87 | 5 | communal | Yes | 1st | Treatment | 2 | 3 | 3 | 4 | Yes | 1 |  |  |  |
| 88 | 5 | communal | Yes | 1st | Treatment | 2 | 2 | 3 | 4 | Yes | 2 |  |  |  |
| 89 | 5 | communal | Yes | 1st | Treatment | 2 | 1 | 3 | 2 | Yes | 1 |  |  |  |
| 90 | 5 | communal | Yes | 1st | Treatment | 2 | 2 | 2 | 4 | Yes | 3 |  |  |  |
| 91 | 5 | communal | Yes | 1st | Treatment | 1 | 2 | 1 | 4 | Yes | 2 |  |  |  |
| 92 | 5 | communal | Yes | 1st | Treatment | 2 | 3 | 1 | 3 | Yes | 1 |  |  |  |
| 93 | 5 | communal | Yes | 1st | Treatment | 2 | 3 | 1 | 4 | Yes | 1 |  |  |  |
| 94 | 5 | communal | Yes | 1st | Treatment | 2 | 3 | 1 | 4 | Yes | 1 |  |  |  |
| 95 | 5 | communal | Yes | 1st | Treatment | 2 | 3 | 1 | 4 | Yes | 3 |  |  |  |
| 96 | 5 | communal | Yes | 1st | Treatment | 2 | 1 | 1 | 3 | Yes | 1 |  |  |  |
| 97 | 5 | communal | Yes | 1st | Treatment | 2 | 1 | 2 | 4 | Yes | 1 |  |  |  |
| 98 | 5 | communal | Yes | 1st | Treatment | 2 | 1 | 3 | 4 | Yes | 1 |  |  |  |
| 99 | 5 | communal | Yes | 1st | Treatment | 1 | 2 | 1 | 3 | Yes | 2 |  |  |  |
| 100 | 5 | communal | Yes | 1st | Treatment | 2 | 3 | 3 | 4 | Yes | 3 |  |  |  |

| Keys | | | |
| --- | --- | --- | --- |
| villages | | Drug administered by | |
| 1 | Borer-4 | 1 | self |
| 2 | Borer-5 | 2 | Gvt. Vet.Clinician |
| 3 | WuhaLimat | 3 | Pvt. Clinician |
| 4 | Misreta |  |  |
| 5 | Wolaita |  |  |
| Drug source | | Source that provide more effective drug | |
| 1 | Gvt. Vet. Clinic | 1 | Gov. vet. Clinic |
| 2 | PVt. Drug store | 2 | PVt. Drug store |
| 3 | open market | 3 | open market |
| Type of drug | | Treatment Frequency/animal/year | |
| 1 | Brown (ISM) | 1 | 1 to 3 |
| 2 | Yellow (DA) | 2 | 4 to 6 |
|  |  | 3 | 7 to 9 |
|  |  | 4 | >9 |
